# Dataset of vehicle chase measurements in real-world subfreezing winter conditions

**DOI:** 10.1016/j.dib.2024.110481

**Published:** 2024-04-26

**Authors:** Ville Leinonen, Miska Olin, Sampsa Martikainen, Ukko-Ville Mäkinen, Santtu Mikkonen, Panu Karjalainen

**Affiliations:** a Department of Technical Physics, University of Eastern Finland, 70211 Kuopio, Finland; b Aerosol Physics Laboratory, Tampere University, 33014 Tampere, Finland; c now at: Department of Atmospheric Sciences, Texas A&M University, College Station, TX, USA; d Department of Environmental and Biological Sciences, University of Eastern Finland, 70211 Kuopio, Finland; e Institute for Advanced Study, Tampere University, 33014 Tampere, Finland

**Keywords:** Vehicle emissions, Cold start, Emission factor, Particle emissions, Particle size distribution

## Abstract

This dataset comprises thorough measurements of light-duty vehicles emissions conducted in Siilinjärvi and Kuopio, Finland, during February 2021, using a mobile laboratory. The measurements focused on subfreezing conditions to capture emissions nuances during cold weather. Measurements were carried out on minimally trafficked roads to diminish external disturbances. The dataset includes a large number of variables from gas and particle emissions. Gaseous emissions of CO, CO_2_, and NO_x_ were measured. Measured variables of particle emissions were number concentration (CPC), size distribution (ELPI+), black carbon concentration (AE33), and chemical composition (SP-AMS).

A total of six light-duty vehicles were investigated, featuring three diesel and three gasoline engines. The measurements incorporated three distinct drive scenarios: subfreezing–cold start, preheated–cold start (utilizing either electrical or fuel-operated auxiliary heaters), and hot start (where a vehicle engine has reached the optimal temperature through prior driving). Each drive type was replicated twice, resulting in six driven rounds per vehicle and 36 rounds in total. Additionally, daily background measurements were conducted by following the same route without chasing a specific vehicle. Meteorological conditions during the measurements were representative of winter in Finland, with outside temperatures ranging from –9 °C to –28 °C. The effect of weather conditions on the measurements were minimal. Only a minor effect was due to the occasional snowfall, especially on the last day when the road surface was snowy, and the car being chased lifted the snow from the road surface. We didn't recognize other factors, such as high wind speeds or major road dust emissions, that could have affected the measurement results.

This dataset serves as a valuable resource for comparing emissions under diverse environmental conditions, particularly in real-life winter settings where data are scarce. Furthermore, it provides an opportunity for meta-analysis of emission factors from various passenger vehicle types. The dataset's richness and specificity make it a valuable contribution to the understanding of winter-time vehicular emissions.

Specifications Table

**Table:** 

Subject	Environmental Science
Specific subject area	Passenger vehicle emissions in subfreezing winter conditions
Data format	Raw
Type of data	Table, Figure, Video
Data collection	The dataset [1] consists of chase measurements from passenger vehicles in subfreezing winter conditions, studying the effect of engine preheating on emissions. The vehicles have been chased with a mobile laboratory; i.e. real-world emissions of vehicles have been measured. The dataset contains measurements of particulate matter, consisting of particle number (CPC) and size (ELPI+), black carbon (AE33), and chemical composition of primary organic aerosol (SP-AMS). In addition, representative gaseous compounds (CO2, NOx) have been measured. On-board diagnostics data from the vehicles and other data related to experiments have been recorded.
Data source location	Measurement Location: Siilinjärvi and Kuopio municipalities, Northern Savonia, Finland Approximate start point of measurements: Latitude 62.982245 degrees north, Longitude 27.732939 degrees east. Data storage affiliation: Tampere University, Tampere, Pirkanmaa, Finland
Data accessibility	Repository name: Zenodo Data identification number: 10.5281/zenodo.10851868 Direct URL to data: https://doi.org/10.5281/zenodo.10851868
Related research article	Olin M., Leinonen V., Martikainen S., Mäkinen U.-V., Oikarinen H., Mikkonen S., Karjalainen P. (2023), Engine preheating under real-world subfreezing conditions provides less than expected benefits to vehicle fuel economy and emission reduction for light-duty vehicles, Applied Energy, 351, 121805, https://doi.org/10.1016/j.apenergy.2023.121805

## 1 Value of the Data

• This dataset provides new insight on passenger vehicle emissions in subfreezing conditions, as well as for studying new methods to estimate vehicle emissions.
• Researchers and other developers may benefit from this dataset, as the dataset is large and provides detailed measurement procedures (raw datasets, metadata, example videos). Thus, the dataset enables both the basic research, but also the development and validation of new data analysis methods.
• The dataset has multiple opportunities for further research. The dataset can be combined with other vehicle emission datasets to provide knowledge about emissions of (passenger) vehicles. The dataset would be available for comparing emissions in different seasons of the year and for meta-analysis. The multivariate analysis of vehicle emission dataset can study the dependence of each emission component on each other, or the dependence of the component on the driving parameters (e.g. on-board diagnostics parameters). The additional video material makes the dataset and measurements conditions easier to understand.

## 2 Background

The aim behind data was to measure both the effect of cold weather and engine preheating to the emissions of light-duty diesel and gasoline vehicles. There are not many studies in the literature that have examined the emissions of vehicles and the effect of preheating in cold, subfreezing conditions. Preheating is mostly used to make it easier to start the engine in cold conditions. Preheating can also be used to heat the cabin and windshield before and during driving. The justification for preheating, besides to reduce engine wear in cold starts and comfort from the warm cabin, have been the improved fuel efficiency and reduced emissions.

The measurements have been conducted by chasing the vehicle with the mobile laboratory ATMo-Lab, that have been used in various type emission measurements. ATMo-Lab contained many measuring instruments that obtained different components of both gaseous and particulate emissions from vehicles.

The dataset in this data article includes also various variables that were not used in the analysis of the original research article [2]. The dataset enables further research about e.g. effect of driving conditions, temperature, and vehicle technologies, especially if this data is used as a part of a larger dataset.

## 3 Data Description

The dataset is a set of chase measurements measuring the emissions of passenger vehicles. The measurements have been conducted in typical Finnish winter conditions, temperature being between -9 and -28 °C. The measurements were performed on the same route during four measurement days. There were six passenger vehicles, each involving six rounds of measurements: two subfreezing–cold engine starts, two preheated–cold engine starts, and two hot engine starts were driven. In addition to the measurement rounds, background rounds measuring the background conditions on the measurement route were performed, and control measurements measuring, e.g., filtered air (zero measurements), were performed.

### 3.1 Metadata

Metadata files of the measurement details are provided (see Table 1 for overall picture of the dataset provided). Measurements followed a certain pattern, containing 1-minute idle periods in the beginning and at the end of a round and two 30-second stops simulating traffic lights and intersections. With each vehicle, the measurement rounds were driven at as similar speeds as possible in each measurement round. Dataset measurement_minutes.xlsx contains the information about the vehicle chase measurements, background measurements, and control measurements conducted, including those idles, stops, and interactions. measurement_minutes.xlsx also contains information about possible interruptions of measurement. Examples of these include other vehicles driving near or by the chased vehicle, and residential scale wood combustion alongside the measurement route. These interruptions, that were marked to the measurement minutes, were logged during the chase measurement by a person located in mobile laboratory ATMo-Lab (Aerosol and Trace Gas Mobile Laboratory, Tampere University, [3,4]).

**Table 1:** The entire dataset collected. Variables in squared brackets are key variables that can be used to merge and compare different datasets and metadata. The variable ‘instrument name’ in measurement_variables.xlsx refers to names of the measurement datasets.

Measurement datasets (.mat, .csv) [index, time]	
ae33	airmarEnviron
airmarGps	airmarSpeed
airmarWind	ams
co2	cpc3
cpc10	cpc23
elpiPlus	nox
obd	
Metadata and description of measurement variables (.xlsx)	

measurement_variables (description of variables in measurement datasets) [instrument name]	
measurement_minutes [index, time, vehicle]	
vehicle_info [vehicle]	

The vehicle information about six measured vehicles, containing information collected from Finnish vehicle database Traficom, is presented in the file vehicle_info.xlsx. Table 2 shows part of the file provided. The file contains information about vehicle engine displacement, maximum power, emission class, and reported emissions by a manufacturer for CO_2_, CO, NO_x_, total hydrocarbons, and particles. The reported emission values are either based on WLTP or earlier version (NEDC).

**Table 2:** Information about the studied vehicles. More information about the vehicles, i.e., reported emissions and consumption, can be found from the dataset file vehicle_info.xlsx.

Make and model	Vehicle class	Commissioning day	Fuel	Odometer reading (km)	Engine displacement (cm3)	Maximum net power (kW)	Emission class	Preheater type and power (kW)
Ford Focus 1.0	M1 / Passenger car	21.8.2018	Gasoline	78000	999	74	Euro 6	0.3, electric
Skoda Octavia 1.0	M1 / Passenger car	25.1.2021	Gasoline	1000	999	81	Euro 6	5.0, fuel
Skoda Octavia 2.0	M1 / Passenger car	5.9.2019	Gasoline	21000	1984	140	Euro 6	5.0, fuel
Audi A6 3.0	M1 / Passenger car	30.12.2008	Diesel	236000	2967	176	Euro 5	1.2, electric
Seat Alhambra 2.0	M1 / Passenger car	2.3.2012	Diesel	169000	1968	103	Euro 5	5.0, fuel
VW Transporter 2.0	N1 / Van	30.8.2019	Diesel	36000	1968	75	Euro 6	0.3, electric

In addition to the traditional measurement minutes, on-board videos illustrating the background measurement round (background_round.mp4) and a chase measurement (measurement_round.mp4) are also available. An on-board camera attached to the front window of an ATMo-Lab recorded the videos of the whole measurement campaign, including all measurement rounds.

### 3.2 Measurement Dataset

The measurement dataset is a raw dataset. The raw dataset contains data needed for the analysis of the outcome of a measurement campaign. The dataset has all the data converted to the units typically used in the analysis of such vehicle emissions datasets. That means that the necessary corrections for e.g., dilution in the measurement system for CPCs, have been calculated in the provided datasets and those are mentioned more specifically in the instrument specific sections.

Table 1 shows the illustration of the entire measurement dataset. Table 3, Table 4, Table 5, Table 6, Table 7, Table 8, Table 9 (presented in section “Experimental design, materials and methods”) describe all the measurement instruments presented in Table 1. For each measurement dataset, a MATLAB data (.mat) and a .csv file are provided, containing the same information about the measurement. The .mat file is the original data format used to store the data, and .csv have been provided for easier accessibility to the data for persons that do not have access to MATLAB.

**Fig. 1:**
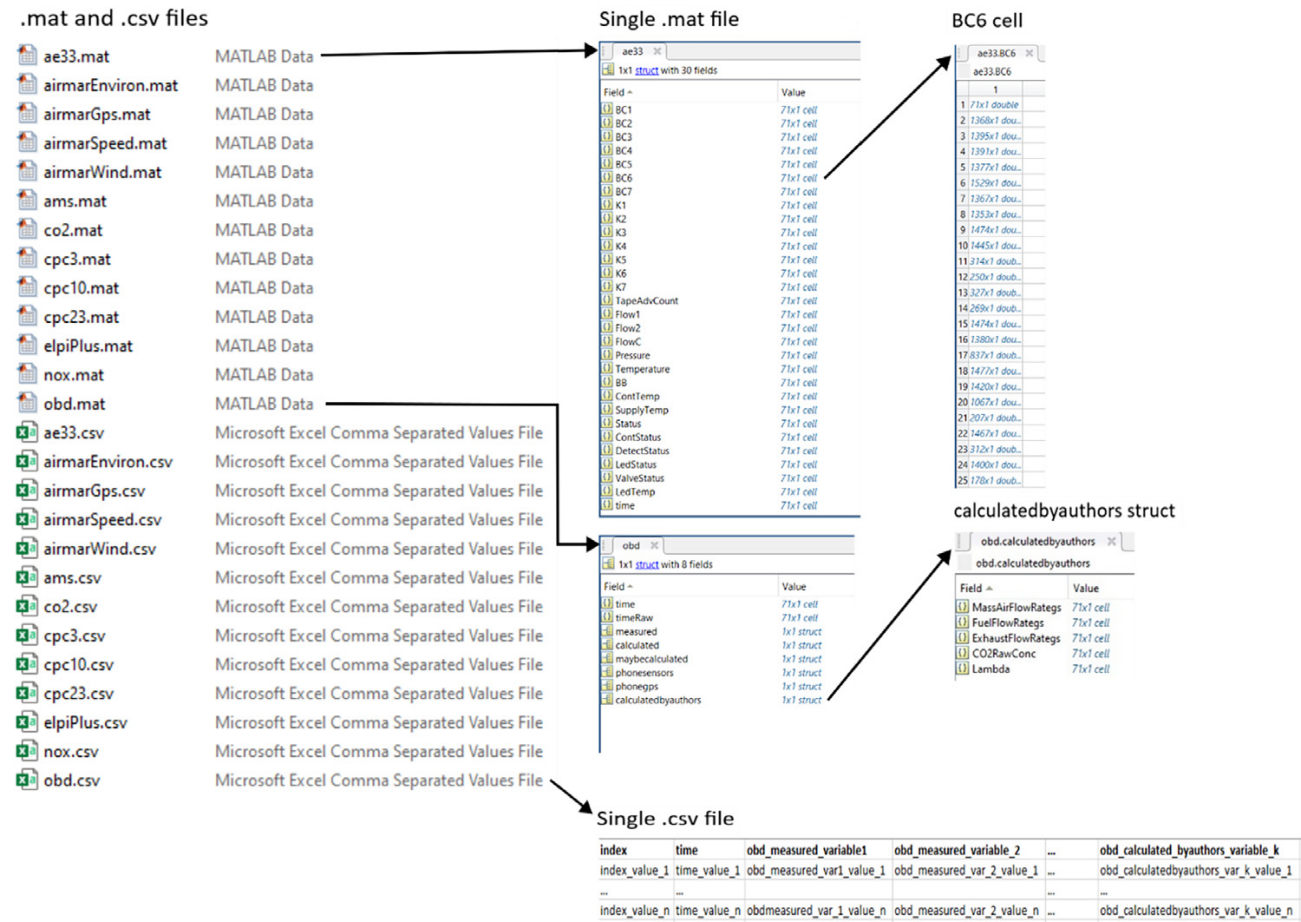
Illustration of files provided from measurement instruments. The figure also shows the structure of .mat and .csv files. The structure is described more in detail in the subsection “Measurement dataset”.

The illustration of the structure of the data files (.mat and .csv) are presented in Fig. 1. Each .mat file contains a struct-type of object that contains variables measured by an instrument as cell-objects. Each cell contains every measurement (chase, background, and control measurements) of a variable as a vector (double). Number (row index in a cell) of a measurement corresponds to an index in the file measurement_minutes.xlsx. In each struct, there is a key variable time that corresponds to a time value in other files. The only exception among .mat files is obd.mat that has an extra layer of structs inside the file (see Fig. 1). The layer is showing the difference of collection type in variables recorded. The reason for the different structure in OBD dataset is that we wanted to emphasize the difference between the information sources of the variables in OBD dataset (e.g. from OBD port, calculated based on other variables, or from phone). The sources are described more in detail in section ‘OBD’. In .csv files, all variables are stored in one file (Fig. 1). The name of the variable in .csv file corresponds to the name in .mat file. For example, the BC6 variable from ae33.mat file is named as ae33_BC6 in ae33.csv file. Each .csv file contains master time (variable time) and measurement index (variable index) as key variables.

In addition to the data files, the file measurement_variables.xlsx contains description about measured variables by instrument for which .csv and .mat files are provided. The information provided in the measurement_variables.xlsx is also provided in Table 3, Table 4, Table 5, Table 6, Table 7, Table 8, Table 9 in the section “Experimental design, materials and methods” below.

## 4 Experimental Design, Materials and Methods

### 4.1 Experimental Design

During planning of the measurement campaign, the intention was to measure the vehicle emissions in winter conditions, including the effect of preheating. The conditions during the measurement campaign were representative to the conditions occurring in Finland during the winter months. Although also warmer temperatures occur in wintertime, the conditions represented here are not abnormal in terms of temperature. According to the Finnish meteorological institute, the average temperature in Kuopio, in wintertime (December-February), between 1991-2020, has been -6.9 °C ([5], the webpage only available in Finnish). The yearly averages of the winter temperatures have been between -1.4 and -15 °C. At the nearest weather station from the measurement area, days with the daily average temperatures under -20 °C occur almost every winter.

**Fig. 2:**
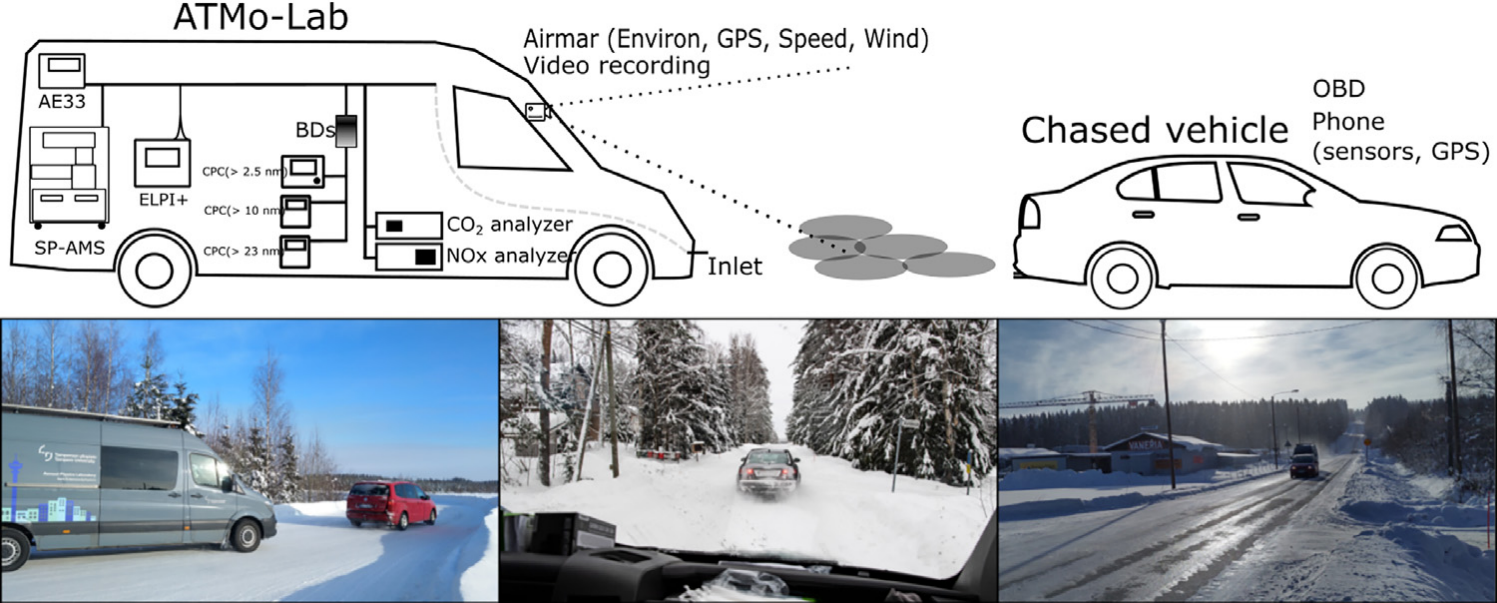
Illustration of the measurement setup. The location (in ATMo-Lab or in the chased vehicle) of measurement instruments and other instruments providing data are illustrated. Below the setup figure, there are photos to illustrate the chase measurement (leftmost and middle photo) and the environment and the conditions during the time of the measurement campaign (rightmost photo). The Figure is modified from the Fig. 2 presented in [2]. The names of the instruments in the top figure are corresponding to the names used in the text. BDs denote two bifurcated-flow diluters used to dilute the sample (i.e. decrease the particle concentration) before CPC instruments.

The dataset is collected by chase measurements, where passenger vehicle is chased by a mobile laboratory ATMo-Lab. Passenger vehicle emission is collected into the sampling inlet from where the emission moves to the measurement instruments via sampling line. Fig. 2 illustrates the sampling system and the idea of chase measurements.

Six light-duty vehicles were measured. The vehicles include three gasoline and three diesel vehicles. The most important technical properties of the vehicles are described in Table 2 (more variables in the dataset, file vehicle_info.xlsx). The effect of preheating the engine was investigated by preheating the engine before some of the drives (marked as ‘Drive route preheated-cold' in the measurement_minutes.xlsx), and not using preheater before some of the drives (marked as ‘Drive route subfreezing-cold'). After some of the subfreezing-cold and preheated-cold drives, another lap was driven with an already warm engine (marked as ‘Drive route hot’). If the hot round does not start within some minutes after the previous round, the engine of the measured vehicle was heated with more intense driving on the highway near the measurement route, to ensure that the normal operating temperature of the engine has been reached before starting the hot round. An example of this kind of heating is marked as ‘Highway chase (engine warming)’ for VW Transporter. At that time, the ATMo-Lab was following the chased vehicle to the highway and there is also the chase data available in the dataset. During other heatings, the ATMo-Lab was waiting near the starting point of the next measurement and hence the heating is not marked explicitly in the measurement_minutes.xlsx.

The corrections made for each dataset have been listed under the section related to the measurement instrument. Time interpolation have been done for all the instruments, so that there is a time point for every second during measurement rounds. SP-AMS didn't measure the data for every second (SP-AMS) and other instruments were missing some time points. The fraction of missing data varied between instruments. For instruments which more than 0.1 % of total time points were originally missing and then interpolated, the indicator variable for interpolation have been provided (Airmar, CPCs, and NO_x_ analyzer). Interpolation means here that no measured values exist in the 1 second time frame. If two values have been measured for during one second time frame, the interpolation of those into the rounded time point (dd-mm-yyyy HH:MM:SS) haven't been considered as interpolation. Multiple values measured during one second time frame is not common in the whole dataset; occurrence is under 1 % for each instrument. The only exception is Airmar, for which gps, speed, wind have usually (> 90 % of the time points) more than one measurement/second and for environmental parameters, the occurrence of multiple values measured during one second time frame is roughly 4 %.

Time synchronization between measurement instruments have been also performed and is not mentioned separately for each instrument. All the instrument times have been corrected to correspond the times of measurement_minutes.xlsx.

### 4.2 Measurement Instruments

Measurement instruments used in the campaign are regularly maintained and calibrated. The last calibrations or maintenance for the instruments were performed 2 months (AE33, CPCs, ELPI+, CO_2_) or one month (NO_x_) before the campaign. The AMS was calibrated 1 week after the campaign. Other instruments (Airmar, OBD reader) were not calibrated. In addition to calibrations, the zero measurements were made for the instruments by sampling outside air through HEPA filter. Zero measurements are listed in the measurement_minutes.xlsx and the measurement data of those are provided in .mat and .csv datasets. Operation of the instruments were also monitored during the campaign by looking at the data recorded. The single ion strength of the AMS was measured every day to detect any drifting.

### 4.3 Aethalometer (AE33)

The dual-spot aethalometer AE33 (Aerosol Co., Slovenia; 5.0 l/min, [6]) measured black carbon (BC). The instrument measures optical attenuation from two filters. The resulted data includes attenuation for seven channels with different wavelengths (370, 470, 520, 590, 660, 880, and 950 nm). The optical attenuation was then changed to BC concentration. In the dataset, the BC concentration for each channel is provided, namely variables BC1 to BC7, variables respective to the wavelengths from shortest to longest. In addition, the compensation parameters (k, for details, see Drinovec et al. (2015) [6]) are provided for each channel, namely variables K1 to K7. The parameters related to operation of the instrument (i.e., advances of the filter tape used in the instrument, air flow parameters, temperature, and internal status codes) are also provided. See Table 3 for a full list of variables and their description.

**Table 3:** List and descriptions of variables provided by the dual-spot aethalometer AE33 (in files ae33.mat and ae33.csv).

Variable	Description
BC1-7	BC calculated from measurements for channel 1-7 (370, 470, 520, 590, 660, 880, and 950 nm, respectively) (ng/m^3^).
K1-7	Compensation parameters for wavelength channels 1-7
TapeAdvCount	Tape advances since start (#)
Flow 1-2, FlowC	Measured flow in spot 1 (Flow1), through the optical chamber (FlowC), and the difference between those two (Flow2) (ml/min). See the manual [7] for more details.
Pressure	Pressure used to report flow (Pa)
Temperature	Temperature used to report flow (°C)
BB	Estimated percentage of BC created by biomass burning (%)
ContTemp	Control board temperature (°C)
SupplyTemp	Supply board temperature (°C)
Status	Internal status code for the instrument
ContStatus	Internal status code for the instrument
DetectStatus	Internal status code for the instrument
LedStatus	Internal status code for the instrument
ValveStatus	Internal status code for the instrument
LedTemp	Internal status code for the instrument
Time	Master time of measurements (dd-mm-yyyy HH:MM:SS)

### 4.4 CPC

CPC battery containing 3 CPCs measured the number concentration particles with instrument cut-off diameters of 2.5, 10 and 23 nm, named as cpc3, cpc10 and cpc23 in the data files. A CPC has an optical counter measuring particles, that are large enough, and that are optically counted based on laser scattering. For more detailed description of CPC operation, see e.g. [8]. The CPCs were CPC 3756 (TSI Inc.) with the nominal cut-off size of 2.5 nm, CPC A20 (Airmodus Ltd) with the nominal cut-off size of 10 nm, and CPC A23 (Airmodus Ltd) with the nominal cut-off size of 23 nm. Cut-off diameter is a diameter that 50 % of the particles with that size are detected (and for larger particles than cut-off diameter, the detection probability is higher). Due to the losses of particles in the sampling lines and in bifurcated-flow diluters (marked as BDs in Fig. 2), the observed samples of particles have higher diameters than reported cut-off sizes of the instruments [2], namely 6.7, 12 and 23 nm for CPCs with nominal cut-off sizes 2.5, 10, and 23 nm, respectively. No specific corrections for sample line losses and particle losses in diluters have been made. In addition to number concentrations, CPCs with cut-off diameters of 10 and 23 nm provided also operating parameters of the instrument, such as air flow, dead time (i.e., time when the particle is between the laser and optical sensor), and number of pulses (of scattered light) detected. Those variables are also provided in the dataset. Concentrations of CPCs have been multiplied with the dilution factors from bifurcated-flow diluters (total dilution factor for the CPC battery is 158). Calibrated detection efficiency of each CPC hasn't been considered in the dataset; coefficients can be found from code script EF_example.m. Table 4 summarizes the variables provided by each instrument.

**Table 4:** List and descriptions of variables provided by the CPC instruments (in files cpc3.mat, cpc10.mat, cpc23.mat, cpc3.csv, cpc10.csv, and cpc23.csv).

	Variable	Description
**cpc10/ cpc23**		
	NominalFlowConc	Concentration based on nominal flow of the instrument (#/cm^3^, in the sampled air, no sample line dilution factor corrected)
	FlowCorrectedConc	Concentration based on measured flow of the instrument (#/cm ^3^, in the sampled air, no sample line dilution factor corrected)
	DeadTime	Dead time of the instrument during the averaging time (µs)
	Npulses	Number of pulses detected during averaging time (#)
	AveragingTime	Time period used to calculate values of other variables (s)
	NominalFlow	Nominal flow used in the calculations (lpm)
	MeasuredFlow	Measured flow (lpm)
	conc	Number concentration of particles (#/cm^3^, dilution in sample lines corrected, FlowCorrectedConc*158)
	time	Master time of measurements (dd-mm-yyyy HH:MM:SS)
	timeinterpolatedcpc10/timeinterpolatedcpc23	Variable that indicates if timepoint is measured or interpolated (0 = measured, 1 = interpolated)
**cpc3**		
	conc	Number concentration of particles (#/cm^3^, dilution in sample lines corrected, dilution factor = 158)
	time	Master time of measurements (dd-mm-yyyy HH:MM:SS)
	timeInterpolatedcpc3	Variable that indicates whether timepoint is measured or interpolated (0 = measured, 1 = interpolated)

The CPC data was mostly measured approximately 1 second resolution. However, the data files produced by CPCs had missing measurements from some time points. Those time points were linearly interpolated, so that the data provided have 1 second time resolution. For cpc3 data, approximately 0.01 % of the data were interpolated. For cpc10 and cpc23, the proportion of interpolated data were significantly larger, namely 22.13 % for both cpc10 and cpc23. Interpolated data have been indicated with variables timeInterpolatedcpc3/10/23, where the name of the variable refers to the instrument cut-off diameters.

### 4.5 ELPI+

Electrical Low Pressure Impactor (ELPI+, Dekati Ltd.) measured the size distribution of emitted particles (see Table 5). The measurement is based on unipolar charging of particles and classification of particles by size in cascade impactor. The number/mass of particles deposited on each state of the cascade impactor is detected by measuring electric current of each stage [9]. The currents are converted to the units of particle number and mass. ELPI+ dataset contains the electric currents in each state and currents after zero correction, diffusion correction, and after smoothing the time series (variables current, currentZeroCorrected, currentZeroCorrectedDiffCorrected, and currectZeroCorrectedDiffCorrectedSmoothed), the number-size distribution (dndlogdp), the mass-size distribution (dmdlogdp), the total number (totConc) and mass (massTot) of the particles. The number-size and mass-size distribution, total number and total mass are calculated from the diffusion corrected electric currents (variable currentZeroCorrectedDiffCorrected). ELPI+ measured total of 14 particle size bins, with size bin median particle diameter between 9.8nm and 7.3 µm. For zero and diffusion correction of ELPI+ data, see Järvinen et al. [9].

**Table 5:** List and descriptions of variables provided by the ELPI+ instrument (in files elpiPlus.mat and elpiPlus.csv).

Variable	Description
current	Electric currents of each impactor stage (fA)
currentZeroCorrected	Electric currents of each impactor stage with adjusted zero levels(fA)
currentZeroCorrectedDiffCorrected	Electric currents of each impactor stage, with adjusted zero levels and diffusion correction (fA)
currentZeroCorrectedDiffCorrectedSmoothed	Electric currents of each impactor stage, with adjusted zero levels, diffusion correction, and smoothed time series (fA)
dpMean	Mean diameters of size bins measured by the instrument (m)
dndlogdp	Number size distribution of particles (dN/dlogdp #/cm^3^)
totConc	Total number concentration of particles (#/cm^3^)
dmdlogdp	Number size distribution of particles (dm/dlogdp µg/cm^3^)
massTot	Total mass concentration of particles (µg/cm^3^)
dM	Distribution of mass to different size bins (µg/cm^3^)
time	Master time of measurements (dd-mm-yyyy HH:MM:SS)

### 4.6 Gas Monitors

Gas emission measurements included CO_2_ (reported in ppm) and NO_x_ (NO_x_ = NO + NO_2_, reported in ppm). CO_2_ (and water) concentration were measured with infrared gas analyzer (LI-840 A, LI-COR Inc.). Infrared measurement is based on the difference ratio in the infrared absorption between two absorption bands, determined separately for CO_2_ and water. In addition to CO_2_ (ppm) and water concentration (ppm,°C), infrared absorptions for both CO_2_ and water are also reported. NOx was measured with chemiluminescence analyzer (Model T201, Teledyne Technologies Inc.). For NO_X_ analyzer, the NO_x_ (NO + NO_2_) concentration was measured. The time resolution of NO_x_ measurement instrument (Model T201, Teledyne Technologies Inc.) was 30 seconds, however the instrument provided measurement value for every second. As the measurement data from other instruments was mostly reported with one second time resolution, the original 30 s resolution data (variable conc) was deconvolved using the Richardson–Lucy deconvolution method with 100 iterations. For each iteration, the result from the previous iteration was used as the initial state for the convolution function. The result of the convolution iterations was then averaged to 10 second resolution. Finally, the volumetric concentration of NO_x_ (ppb) was converted to mass concentration (µg/m^3^) by assuming a constant NO_2_/NO_x_ ratio for the whole campaign (25 %) based on the earlier study by Carslaw et al. [10] (variable deconvolvedConc). I.e. the volumetric NO_x_ concentration was assumed to be 75 % of NO and 25 % of NO_2_. For NOx, around 5.99 % of the time points were not measured, and for those, the values of each variable were interpolated to the dataset. The variable timeInterpolatednox indicates the interpolated time points. See Table 6 for the description of variables measured by gas monitors.

**Table 6:** List and descriptions of variables provided by gas monitors for CO_2_ (LI-840A, in files co2.mat and co2.csv) and NO_x_ (Model T201, in files nox.mat and nox.csv).

	Variable	Description
**co2**		
	co2Ppm	CO_2_ concentration measured by CO_2_ instrument (ppm)
	h2oPpt	H_2_O concentration measured by CO_2_ instrument (ppt)
	h2oC	H_2_O concentration (°C)
	co2_absorption	IR absorptance ratio between 4.26 µm and 3.95 µm (reference) centered filter
	h2o_absorption	IR absorptance ratio between 2.595 µm and 2.35 µm (reference) centered filter
	time	Master time of measurements (dd-mm-yyyy HH:MM:SS)
		
n**ox**		
	conc	Gas concentration of NOx measured by instrument (ppb)
	deconvolvedConc	Gas concentration of NOx after Richardson-Lucy deconvolution and 10 second averaging (see subsection “Gas monitors” for details) (µg/cm^3^)
	time	Master time of measurements (dd-mm-yyyy HH:MM:SS)
	timeInterpolatednox	Variable that indicates whether timepoint is measured or interpolated (0 = measured, 1 = interpolated)

### 4.7 SP-AMS

The soot-particle aerosol mass spectrometer (SP-AMS) measured the chemical composition of aerosol particles. All variables are described in Table 7. Detailed description of the data analysis tools and steps are given in [2]. The measurements are reported as a mass of six species: organic, sulfate, nitrate, ammonium, chloride (all non-refractory), and refractory black carbon. In addition to mass concentrations of those species, ratios of oxygen to carbon (O:C), hydrogen to carbon (H:C), and organic matter to organic carbon (OM:OC) in particles were measured. The oxidation state of particles and total mass of particles are also reported. The SP-AMS data have been interpolated to one-second time resolution using linear interpolation. The original time resolution was 22 seconds. Variable timeInterpolatedams shows the measured and interpolated time points.

**Table 7:** List and descriptions of variables provided by AMS (in files ams.mat and ams.csv).

Variable	Description
HROrg_M	Mass of non-refractory organic species (µg/m^3^)
HRSO4_M	Mass of non-refractory sulfate species (µg/m^3^)
HRNO3_M	Mass of non-refractory nitrate species (µg/m^3^)
HRNH4_M	Mass of non-refractory ammonium species (µg/m^3^)
HRChl_M	Mass of non-refractory chloride species (µg/m^3^)
HRBC_M	Mass of refractory black carbon species (µg/m^3^)
Ratio_O_C	O:C ratio measured
Ratio_H_C	H:C ratio measured
Ratio_OM_OC	OM:OC ratio measured
Osc	Oxidation state measured
massTot	Total mass of particles measured by the instrument (µg/m^3^)
time	Master time of measurements (dd-mm-yyyy HH:MM:SS)
timeInterpolatedams	Variable that indicates whether timepoint is measured or interpolated (0 = measured, 1 = interpolated)

During the first two days, SP-AMS measured fresh aerosol, and during the last two days, aged aerosol (after treatment of Tampere secondary aerosol reactor, TSAR, [11]) was measured. In this dataset, the AMS data is provided from the first two days of the measurement campaign (fresh aerosol). The analysis of aged aerosol also requires knowledge about the operating conditions of the aging reactor (e.g., OH concentration, photochemical age). For reasoning providing only fresh aerosol data, see also discussion related to aged aerosol in section Limitations.

### 4.8 Airmar Weather Station

Airmar weather station measured the weather and location on the top of the ATMo-Lab during the measurements. Table 8 shows all the variables measured by the weather station. Weather variables measured by Airmar were temperature, relative humidity, air pressure, wind angle, and wind speed. However, with wind speed and angle, the measurements that were performed during driving were affected by the moving vehicle. The location variables measured were latitude, longitude, altitude, and speed of the ATMo-Lab. Altitude was both measured by Airmar, and as the measurements contain some uncertainty, the correct altitude for each latitude-longitude pair was retrieved from database of National Land survey of Finland (variable altitudeMML). The retrieved altitude parameter still has some uncertainty, that is due to the uncertainty of the latitude and longitude measurements of the weather station.

**Table 8:** List and descriptions of variables provided by Airmar weather station (in files airmarEnviron.mat, airmarEnviron.csv, airmarGPS.mat, airmarGPS.csv, airmarSpeed.mat, airmarSpeed.csv, airmarWind.mat, and airmarWind.csv).

Variable	Description
**airmarEnviron**	
temperature	Outdoor temperature (°C)
rh	Outdoor relative humidity (%)
pressure	Air pressure (hPa)
time	Master time of measurements (dd-mm-yyyy HH:MM:SS)
timeInterpolatedairEnvPar	Variable that indicates whether timepoint is measured or interpolated (0 = measured, 1 = interpolated)
**airmarGPS**	
timeGPS	Time given by Airmar GPS (dd-mm-yyyy HH:MM:SS)
latitude	Latitude coordinate (N degrees with decimals)
longitude	Longitude coordinate (E degrees with decimals)
altitude	Altitude given by Airmar GPS (meters above sea level)
altitudeMML	Altitude derived based on latitude and longitude coordinates, from database of National Land survey of Finland (meters above sea level)
time	Master time of measurements (dd-mm-yyyy HH:MM:SS)
timeInterpolatedairGPS	Variable that indicates whether timepoint is measured or interpolated (0 = measured, 1 = interpolated)
**airmarSpeed**	
speed	Speed of ATMo-Lab (m/s)
time	Master time of measurements (dd-mm-yyyy HH:MM:SS)
timeInterpolatedairSpeed	Variable that indicates whether timepoint is measured or interpolated (0 = measured, 1 = interpolated)
**airmarWind**	
windSpeed	Wind speed on top of the ATMo-Lab (including air movement caused by driving, m/s)
windAngle	Wind angle (degrees with respect to the ATMO-Lab, no major protection from airflow caused by driving)
time	Master time of measurements (dd-mm-yyyy HH:MM:SS)
timeInterpolatedairWind	Variable that indicates whether timepoint is measured or interpolated (0 = measured, 1 = interpolated)

### 4.9 OBD

OBD dataset contains variables logged with the Bluetooth OBD sensor from the OBD port of the chased vehicle. The sensor sent the data to a mobile phone located in the chased vehicle, near the sensor. The variables given by OBD sensor were directly measured from the vehicles’ OBD port (total of 87 variables, obd.measured in .mat files, obd_measured in .csv:s), calculated by the OBD sensor based on measured data (12 variables, obd.calculated), the variables that we couldn't define whether those were measured or calculated by OBD sensor (11 variables, obd.maybecalculated), the variables that were measured by phone used to log the OBD data (14 variables by phone sensors, obd.phonesensors + 6 from the phone GPS, obd.phonegps), and calculated by the authors based on other OBD data (5 variables, obd.calculatedbyauthors). Variables calculated by authors contain variables measured by the OBD sensor that were not fully available for all the vehicles from the measured and calculated OBD dataset. Variables were important when determining the emission factors of the vehicles and were hence calculated for all the vehicles. These variables were mass air flow rate, fuel flow rate, exhaust flow rate, and raw CO_2_ concentration for all vehicles, and lambda parameter for diesel vehicles. A complete list of OBD variables and their description can be found from Table 9.

**Table 9:** List and descriptions of variables provided by OBD sensor and phone logging the OBD data (in files obd.mat, and obd.csv).

Variable	Description
time	Master time of measurements (dd-mm-yyyy HH:MM:SS)
timeRaw	Time measured by OBD logger (dd-mm-yyyy HH:MM:SS)
**measured**	
VehicleSpeedkmh	Speed of chased vehicle (km/h)
FuelSystem1Status	Fuel system status of system 1 (see https://en.wikipedia.org/wiki/OBD-II_PIDs)
FuelSystem2Status	Fuel system status of system 2 (see https://en.wikipedia.org/wiki/OBD-II_PIDs)
CalculatedLoadValue	Calculated engine load (%)
EngineCoolantTemperatureC	Engine coolant temperature (°C)
ShortTermFuelTrimBank1	Short term fuel trim bank (%, see https://en.wikipedia.org/wiki/OBD-II_PIDs)
LongTermFuelTrimBank1	Long term fuel trim bank (%, see https://en.wikipedia.org/wiki/OBD-II_PIDs)
IntakeManifoldAbsolutePressurekPa	Intake manifold absolute pressure (kPa)
EngineRPMRPM	Engine rpm (rounds per minute)
IgnitionTimingAdvanceFor1Cylinderdeg	Ignition timing advance for cylinder (degrees)
IntakeAirTemperatureC	Intake air temperature (°C)
MassAirFlowRategs	Mass air flow rate (g/s)
AbsoluteThrottlePosition	Absolute throttle position (%)
LocationOfOxygenSensors	Location of oxygen sensors
O2VoltageBank1Sensor2V	Voltage of the sensor (V)
ShortTermFuelTrimBank1Sensor2	Short term fuel trim bank (%, see https://en.wikipedia.org/wiki/OBD-II_PIDs)
OBDRequirementsToWhichVehicleOrEngineIsCertified	Index of showing whether vehicle or engine is certified (6)
TimeSinceEngineStartsec	Time since engine start (seconds)
FuelRailPressurekPa	Fuel rail pressure (kPa)
O2SensorLambdaBank1Sensor1	Indicatior value for O2 Lambda sensor (for diesel vehicles)
O2SensorVoltageWideRangeBank1Sensor1V	O2 sensor voltage (V)
CommandedEGR	Commanded EGR (%)
EGRError	EGR error (%)
CommandedEvaporativePurge	Commanded evaporative purge (%)
FuelLevelInput	Fuel tank level (%)
NumberOfWarmupsSinceDTCsCleared	Number of warmups since DTCs cleared (#)
DistanceTraveledSinceDTCsClearedkm	Distance traveled since DTCs cleared (km)
BarometricPressurekPa	Barometric pressure (kPa)
O2SensorLambdaWideRangecurrentProbeBank1Sensor1	Indicator value for the sensor (∼1)
O2SensorCurrentWideRangeBank1Sensor1mA	O2 sensor current (mA)
CatalystTemperatureBank1Sensor1C	Catalyst temperature sensor (°C)
ControlModuleVoltageV	Control module voltage (V)
AbsoluteLoadValue	Absolute load value (%)
FuelAirCommandedEquivalenceRatio	Commanded fuel air equivalence ratio
RelativeThrottlePosition	Relative throttle position (%)
AmbientAirTemperatureC	Ambient air temperature (°C)
AbsoluteThrottlePositionB	Absolute throttle position (%)
AcceleratorPedalPositionD	Accelerator pedal position (%)
AcceleratorPedalPositionE	Accelerator pedal position (%)
CommandedThrottleActuatorControl	Commanded throttle actuator (%)
FuelType	Fuel type (1=gasoline, 0 = not available [=mostly diesel])
AbsoluteEvapSystemVaporPressurePa	Absolute evap. system vapor pressure (kPa)
LongTermSecondaryOxygenSensorTrimBank1	Long term secondary oxygen sensor trim (%)
EngineFuelRatelhr	Engine fuel rate (l/hour)
ActualEnginePercentTorque	Actual engine torgue (%)
EngineReferenceTorqueNm	Engine reference torque (Nm)
AuxiliaryInputsOutputsStatus	Auxiliary input and output status (0-100)
EngineCoolantTemperature1C	Engine coolant temperature (°C)
EngineCoolantTemperature2C	Engine coolant temperature (°C)
CommandedEGRADutyCycleposition	Commanded EGR duty cycle position (%)
ActualEGRADutyCycleposition	Actual EGR duty cycle position (%)
EGRAError	EGRA error (%)
ExhaustGasRecirculationTempSensorABank1Sensor1C	Exhaust gas recirculation temperature (°C)
ExhaustGasRecirculationTempSensorCBank1Sensor2C	Exhaust gas recirculation temperature (°C)
ExhaustGasRecirculationTempSensorBBank2Sensor1C	Exhaust gas recirculation temperature (°C)
ExhaustGasRecirculationTempSensorDBank2Sensor2C	Exhaust gas recirculation temperature (°C)
CommandedFuelRailPressureAkPa	Commanded fuel rail pressure (kPa)
FuelRailPressureAkPa	Fuel rail pressure (kPa)
FuelRailTemperatureAC	Fuel rail temperature (°C)
FuelRailTemperatureBC	Fuel rail temperature (°C)
CommandedBoostPressureAkPa	Commanded boost pressure (kPa)
BoostPressureSensorAkPa	Boost pressure sensor (kPa)
CommandedVariableGeometryTurboAPosition	Commanded variable geometry turbo position (%)
VariableGeometryTurboAPosition	Variable geometry turbo (VGT) position (%)
VGTAControlStatus	VGT control status
ExhaustPressureSensorBank1kPa	Exhaust pressure sensor (kPa)
ChargeAirCoolerTemperatureBank1Sensor1C	Charge air cooler temperature (°C)
ChargeAirCoolerTemperatureBank1Sensor2C	Charge air cooler temperature (°C)
ChargeAirCoolerTemperatureBank2Sensor1C	Charge air cooler temperature (°C)
ChargeAirCoolerTemperatureBank2Sensor2C	Charge air cooler temperature (°C)
ExhaustGasTemperatureBank1Sensor1C	Exhaust gas temperature (°C)
ExhaustGasTemperatureBank1Sensor2C	Exhaust gas temperature (°C)
ExhaustGasTemperatureBank1Sensor3C	Exhaust gas temperature (°C)
ExhaustGasTemperatureBank1Sensor4C	Exhaust gas temperature (°C)
NOxSensorConcentrationBank1Sensor1ppm	NOx concentration (ppm)
NOxSensorConcentrationBank1Sensor2ppm	NOx concentration (ppm)
AverageNOxReagentConsumptionlhr	NOx reagent consumption (l/hour)
AverageDemandedNOxReagentConsumptionlhr	Demanded NOx reagent consumption (l/hour)
NOxReagentTankLevel	NOx reagent tank level (%)
DistanceTravelledInCurrentSCR10KBlock010000Kmkm	Distance travelent in current SCR 10K km block (km)
NormalizedTriggerForDPFRegen	Normalized trigger for DPF regeneration
AverageDistanceBetweenDPFRegenskm	Average distance between DPF regenerations (km)
EngineFrictionPercentTorque	Engine friction torque (%)
EngineFuelRategs	Engine Fuel rate (g/s)
VehicleFuelRategs	Vehicle fuel rate (g/s)
EngineExhaustFlowRategs	Engine exhaust flow rate (g/s)
InputVoltageReadByTheScanToolV	Input voltage read by the scan tool (V)
**calculated**	
InstantFuelEconomyl100Km	Fuel consumption based on current fuel rate (l/100km)
TotalFuelEconomyl100Km	Average fuel consumption in the trip (l/100km)
FuelRatelhr	Current fuel rate (l/hr)
TripDistancekm	Trip distance (km)
TripFuell	Consumpted fuel in the trip (l)
TripFuelEconomyl100Km	Average fuel consumption in the trip (l/100km)
TripDurationmin	Duration of trip from start of an engine (min)
HardBrakeCount	Cumulative number of hard brakings in the trip (#)
HardAccelCount	Cumulative number of hard accelerations in the trip (#)
IdlingCount	Cumulative number of idle periods in the trip (#)
SecondsIdlingsec	Cumulative time of idle time in the trip (#)
MaxSpeedkmh	Maximum speed in the trip so far (km/h)
**maybecalculated**	
IntakeManifoldAbsolutePressurekPa1	Intake manifold absolute pressure (kPa)
MassAirFlowRategs1	Mass air flow rate (g/s)
BoostkPa	Boost pressure (kPa)
Accelerationms	Acceleration (m/s)
AccelerationAvgms	Averaged acceleration (m/s)
EnginePowerhp	Engine power (hp)
EngineTorqueNm	Engine torque (Nm)
AFCommanded	AF commanded
AFActual	AF actual
FuelRemainingl	Fuel remaining (l)
DistanceToEmptykm	Distance to empty fuel tank (km)
**phonesensors**	
AccelXms	Acceleration to X direction (relative to the phone bearing, m/s)
AccelYms	Acceleration to Y direction (relative to the phone bearing, m/s)
AccelZms	Acceleration to Z direction (relative to the phone bearing, m/s)
AccelGravXms	Acceleration to X direction (relative to the phone bearing, m/s) due to gravity
AccelGravYms	Acceleration to Y direction (relative to the phone bearing, m/s) due to gravity
AccelGravZms	Acceleration to Z direction (relative to the phone bearing, m/s) due to gravity
RotationRateXdegs	Rotation to X direction (degrees)
RotationRateYdegs	Rotation to Y direction (degrees)
RotationRateZdegs	Rotation to Z direction (degrees)
Rolldeg	Roll (degrees)
Pitchdeg	Pitch (degrees)
MagnetometerXT	Magnetometer reading to X direction (relative to the phone)
MagnetometerYT	Magnetometer reading to Y direction (relative to the phone)
MagnetometerZT	Magnetometer reading to Z direction (relative to the phone)
**phonegps**	
Latitudedeg	Latitude coordinate from phone (degrees)
Longitudedeg	Longitude coordinate from phone (degrees)
Altitudem	Altitude from phone (m above sea level)
Bearingdeg	Bearing of the phone (degrees)
GPSSpeedkmh	Speed from phone GPS (km/h)
HorzAccuracym	Horizontal accuracy reported by phone (m)
**calculatedbyAuthors**	
MassAirFlowRategs	Engine mass air flow rate (g/s)
FuelFlowRategs	Fuel flow rate (g/s)
ExhaustFlowRategs	Exhaust flow rate (g/s)
CO2RawConc	Raw CO2 concentration in the exhaust (ppm)
Lambda	Air-Fuel equivalence ratio (for diesel vehicles only, 1 = stoichiometric)

### 4.10 Data Processing

The codes for emission factor calculation of CPC battery have been provided in the MATLAB code script EF_example.m. The method calculating the emission factor is the same as in [2]. Shortly, the dilution ratio (DR) between the tailpipe and sampling inlet in ATMo-Lab is calculated in a hybrid way: using the method that is based on the dependence of CO2-based DR:

$$DR_{t}=\frac{CO_{2,raw,t}-CO_{2,bg}}{CO_{2,t}-CO_{2,bg}}$$

when CO2 is produced and using Near-Wake Dilution model introduced in [12] when the vehicle is not producing CO2. Variable t is the measurement time, $CO_{2,raw,t}$ is the raw exhaust (tailpipe) concentration of CO2, $CO_{2,bg}$ is the background CO2 concentration, and $CO_{2,t}$ is the measured CO2 concentration in the ATMo-Lab. The NWD model is based on the (assumed linear) dependence of CO2-based DR from the ratio of speed and exhaust flow rate of the chased vehicle. The slope fitted for NWD model is vehicle specific and the calculation of the slope is presented in the code script. Some additional functions needed to run the code script are also provided. Even though the code script itself presents EF calculation for CPCs, it is not instrument specific and can be modified to be used also for other measurement instruments than CPCs.

## Limitations

OBD dataset was mostly missing during one round of measurement (index 3 in measurement_minutes.xlsx). Some of CO_2_ measurements were missing during another round of measurement (index 33). CPC data of > 23 nm particles were missing from another round of measurement (index 51) and partially from round 3. In addition, some of the variables with name starting ‘OBD_measured_’ are missing for some of the vehicles. Missing values have been marked with ‘NaN’ in .mat files and ‘NA’ in .csv files.

The data of the operation of fuel-operated auxiliary heaters (AHs) during driving is not available, as the vehicles’ OBD systems didn't record such data. It is known that some of the AHs can turn themselves on during driving. Both Skodas and Seat had AH installed.

The data related to the aged aerosol via aging reactor were measured for AMS during the last two days. An ELPI was also measuring aged aerosol. The dataset of aged aerosol requires expertise about the operation and interpretation of operating conditions of the aging reactor. Hence, we decided to leave the data related to aged aerosol out of this dataset. Researchers interested in those measurements can contact the corresponding author and ask about those measurements.

## Ethics Statement

The authors have read and follow the ethical requirements for publication in Data in Brief and confirming that the current work does not involve human subjects, animal experiments, or any data collected from social media platforms.

## CRediT authorship contribution statement

**Ville Leinonen:** Software, Validation, Formal analysis, Investigation, Data curation, Writing – original draft. **Miska Olin:** Conceptualization, Software, Validation, Formal analysis, Investigation, Data curation, Writing – review & editing, Visualization, Supervision. **Sampsa Martikainen:** Conceptualization, Investigation. **Ukko-Ville Mäkinen:** Formal analysis, Data curation, Writing – review & editing. **Santtu Mikkonen:** Conceptualization, Investigation, Resources, Data curation, Writing – review & editing, Supervision, Project administration, Funding acquisition. **Panu Karjalainen:** Conceptualization, Investigation, Resources, Data curation, Writing – review & editing, Visualization, Supervision, Project administration, Funding acquisition.

## Data Availability

Dataset of vehicle chase measurements in real-world subfreezing winter conditions (Original data) (Zenodo).
